# Gamma-irradiated stibnite thin films set a remarkable benchmark performance for photoelectrochemical water splitting

**DOI:** 10.1039/d4ra01382d

**Published:** 2024-04-17

**Authors:** Adel Chihi

**Affiliations:** a Photovoltaic Laboratory, Research and Technology Centre of Energy Borj-Cedria Science, and Technology Park, BP 95 2050 Hammam Lif Tunisia supereagle2791@yahoo.fr

## Abstract

The study sets out to show the positive impact of sulfur vacancy engineering on the structural, morphological, optical, electrical, and photoelectrochemical (PEC) properties of Sb_2_S_3_ films synthesized using the spin coating technique. The produced films were exposed to γ-irradiation with different doses from 0 to 20 kGy. We have demonstrated the formation of sulfur vacancies and loss of oxygen content in the irradiated samples. XRD measurements revealed that all films exhibit a polycrystalline structure, and the crystallite size increases with the rising radiation dose, reaching the highest value of 87.4 nm measured for the Sb_2_S_3_ film irradiated with 15 kGy. The surface roughness of the irradiated samples increases with increasing γ-irradiation dose. The increase in surface roughness not only raises the active sites but enhances the conductivity of the Sb_2_S_3_ material as well. The wettability properties of the irradiated films were affected by γ-irradiation doses and the sample irradiated with 15 kGy exhibited the lowest hydrophobicity compared to others. The Hall measurements reveal that irradiated samples exhibit p-type semiconductor behavior. The optical band gap decreased progressively from 1.78 eV to 1.60 eV up to the irradiation dose of 15 kGy and slightly increased thereafter. The irradiated sample with 15 kGy showed a maximum photocurrent density of *ca.* 1.62 mA cm^−2^ at 0 V *vs.* reverse hydrogen electrode (RHE) under AM 1.5 G illumination with applied bias photon-to-current efficiency (ABPE) of 0.82% at 0.47 V *vs.* RHE, suggesting superior PEC water splitting performance compared to other samples. At 0 V *vs.* RHE and 648 nm, the incident photon current efficiency (IPCE) and absorbed photon current efficiency (APCE) of the photocathode irradiated with 15 kGy are significantly higher than those of the other photocathodes with values of 9.35% and 14.47%, respectively. Finally, Mott–Schottky measurement was also performed on all photocathodes to estimate their acceptor density and flat band potential.

## Abstract 2

The study sets out to show the positive impact of sulfur vacancy engineering on the structural, morphological, optical, electrical, and photoelectrochemical (PEC) properties of Sb_2_S_3_ films synthesized using the spin coating technique.

## Graphical abstract

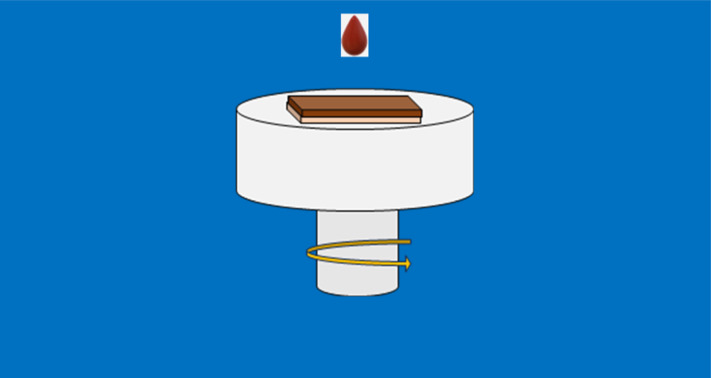

## 1. Introduction

Growing global affluence and population are driving the current surge in interest in renewable energy technology, as the world's energy consumption is predicted to reach over 240 million barrels per day by 2035, combined with depleting fossil fuel sources.^1^ The production of dihydrogen (H_2_) through solar water splitting, referred to as solar-to-hydrogen (STH), serves as the ultimate source of all the energy consumed today. This process provides a sustainable and environmentally friendly energy solution for future generations,^2,3^ playing a crucial role in propelling the transition toward a global carbon-neutral social economy and positioning it as a key facilitator for a greener and more sustainable future.^4^ In this regard, creating clean hydrogen using solar energy has garnered international attention as it presents a viable solution for alleviating the escalating energy costs in developed countries.^5^ To fulfill the sustainable energy purpose, photoelectrochemical (PEC) water splitting is amongst the most promising technologies for STH production to develop renewable energy.^6,7^ Some challenges associated with water-splitting cells include high energy consumption, deterioration of electrodes over time, and the need for catalysts to facilitate the chemical reaction (2H_2_O → 2H_2_ + O_2_). Research is currently underway to address these challenges and improve the efficiency and durability of electrolysis technologies. Among the available solutions, the PEC system stands out as an economically feasible water-splitting cell. It is constructed using cost-effective semiconductor materials, making it a practical choice for this purpose.^8^ Presently, the most reported low-cost photoelectrodes are composed of semiconductors, such as CuIn_*x*_Ga_(1−*x*)_ Se_2_,^9^ CuBi_2_O_4_ (ref. 10), and Cu_2_O.^11^ Among the semiconductors, antimony sulfide Sb_2_S_3_ would be a respectable candidate for use in PEC water splitting^12^ thanks to its economic cost, lacks chemical elements with limited availability or elevated levels of toxicity, including Indium (In), Tellurium (Te), Cadmium (Cd), and Lead (Pb). It also exhibits strong optical absorption (*α* > 10^4^ cm^−1^) in the visible range and near-IR spectral range,^13^ a direct band gap of 1.5–1.7 eV,^14,15^ has one-dimension (1D) parallel nano-ribbon grain structure (Sb_4_S_6_)_*n*_,^16,17^ power conversion efficiency about 7.5%.^18^ In contrast to other semiconductor materials like Si, CdTe, and CuIn_*x*_Ga_(1−*x*)_ Se_2_ (CIGS), Sb_2_S_3_ possesses intrinsically advantageous properties at grain boundaries (GBs). Indeed, they can impede the motion of dislocations, act as barriers to charge carriers, facilitate charge transfer processes, and improve thermal conductivity. These characteristics make GBs crucial in determining the overall properties and performance of Sb_2_S_3_ materials. This inherent quality provides a promising and sustainable solution to the challenges associated with conventional semiconductor absorber materials. In those conventional materials, the disruption of covalent bonds leads to the emergence of defect states and recombination centers along GBs, posing a substantial barrier to charge collection and potentially reducing device efficiency owing to recombination at interfaces. Nevertheless, the Sb_2_S_3_ semiconductor has drawbacks such as electron–hole recombination, native defects (interstitial sulfur (S_i_), vacancies of Sb, and antisite substitutions (S_Sb_, Sb_s_),^19^ what is more, the slow charge transfer kinetics limits the PEC performance and leads to reduced stability of the Sb_2_S_3_ material. In response to these enormous obstacles, scientists have put forward numerous strategies for achieving efficient and practical water splitting of Sb_2_S_3_ semiconductors. Numerous common technologies have been employed, including a protective layer,^20^ morphology control,^21^ doping technique,^22^ cocatalyst material,^23^ and so on. Consistent with previous studies, the introduction of surface defects through γ-ray irradiation represents one of several approaches capable of modifying the structural, optical, and electrical properties of the target material.^24^ Indeed, the surface modification strategy produced by γ-radiation presents a straightforward and cost-effective approach to enhancing PEC water splitting. This strategy offers several advantages, including its superior penetrating power compared to other techniques. Moreover, it can avoid contaminating the target material and generating radioactive by-products. In addition, γ ray irradiation could vary the band gap width (annihilate defect and/or create defects), accelerate the extraction rate of the photogenerated holes, rearrange the charge density, and generate more delocalized electrons, providing highly efficient and stable photoelectrodes for STH generation. So, γ-ray irradiation treatments can induce variations in material properties through a self-regulating process. This unique effect can be harnessed to design materials with desired properties. After γ-ray irradiation, various defects annihilated on the surface and in the bulk of Sb_2_S_3_ due to the cascade collision of irradiated ions and target material. These annihilated defects led to a rise in the electrical conductivity of Sb_2_S_3_ films, which ensured the rapid transport charge carriers and improved the PEC performance. The impact of γ-ray irradiation on the formation, structural, and optical properties of some semiconductor materials such as ZnO,^25^ TeO_2_,^26^ ZnIn_2_S_4_,^27^ and g-C_3_N_4_ films^28^ was also reported to boost the photocurrent density from 3.59 to 5.86 μA cm^−2^ at 1.23 V *vs. E*^0^_Ag/AgCl_, even though decreases the band gap energy from 2.82 to 2.76 eV. Han *et al*. found that the PEC water splitting of Sb_2_S_3_/Sb_2_Se_3_ heterojunction is three times higher than that of Sb_2_Se_3_ and Sb_2_S_3_.^29^ Meanwhile, Wang *et al*. found that doping Sb_2_S_3_ film with the Bi element can control the phase composition and lattice parameters, resulting in a decrease in the optical band gap, a raise in the carrier concentration, and a decrease in the charge transfer resistance, which helps to improve the PEC performance.^30^ Numerous methods have been used to synthesize high-performance Sb_2_S_3_ photoelectrodes, including hydrothermal methods,^31^ chemical bath deposition,^32^ thermal evaporation methods,^33^ and so on. Among the various techniques, the spin coating process stands out for its efficiency and minimal material waste. It is considered an environmentally friendly thin film deposition technique in several industries. To the best of our knowledge, the experimental study of the influence of γ radiation on Sb_2_S_3_ films to improve the PEC water-splitting device has never been carried out. In this work, we investigate the effect of irradiation with γ-ray on the Sb_2_S_3_ semiconductor as an absorber layer grown on an ITO substrate by a fast-turnaround spin coating technique for efficient PEC water splitting. Compared to the morphology of a bare Sb_2_S_3_ thin film, the irradiated films displayed improved surface S vacancies and increased surface-active sites. This improvement helps to attenuate exciton pair recombination arising from deep-level defects in Sb_2_S_3_ material. By optimizing the γ-ray irradiation dose, the Sb_2_S_3_ photoelectrode with better performance was obtained. Besides, the PEC performance after irradiation with γ-ray increases, and the photocurrent density could reach 1.62 mA cm^−2^ at 0 V *vs.* reverse hydrogen electrode (RHE), which is almost 2.7 times higher than the bare one. The improved photoelectrochemical (PEC) performance of irradiated Sb_2_S_3_ films is likely the result of a synergistic effect between increased light absorption and increased concentration of photogenerated carriers.

## 2. Experimental section

### 2.1 Chemicals

Antimony(iii) chloride (SbCl_3_, 97%), thiourea (CH_4_N_2_S, 99% pure), 2-methoxy ethanol (C_3_H_8_O_2_, 98%), ethanol (C_2_H_6_O), acetone (C_3_H_6_O), and hydrochloric acid (HCl) were provided by Sigma-Aldrich company and employed as received without further treatment. The chemical reagents used for the synthesis were of analytical grade. Indium-doped tin oxide (ITO) conductive glass slides with surface resistivity of 10 Ω sq^−1^ and thickness of 2.2 mm were used as substrates for the deposition of Sb_2_S_3_ thin films.

### 2.2 Synthesis of Sb_2_S_3_ photoelectrodes

**Fig. 1:**
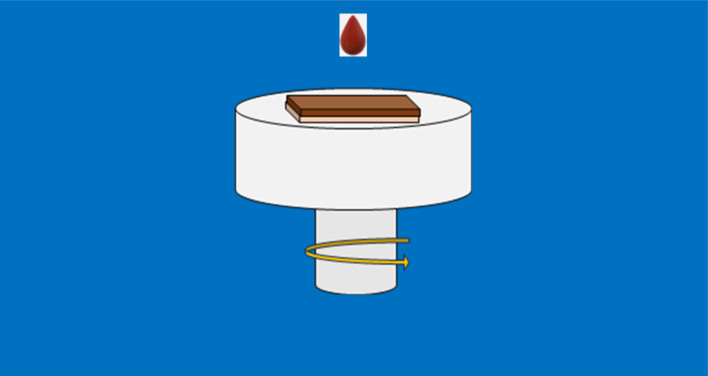
Synthesis protocol for Sb_2_S_3_ thin films.

The solution bath was made up of 1.5 mmol of antimony(iii) chloride and 4 mmol of thiourea as antimony, and sulfur precursors, respectively. Each precursor was dissolved into 10 mL of 2-methoxy ethanol as solvent. After adjusting the pH of the acid bath with a carefully measured drop of HCl, the solution achieved the desired acidic conditions with a pH value of 4.^34^ Afterward, the obtained solution was stirred uniformly under a constant magnetic field followed by heating to 60 °C for 30 min to produce a brown and homogeneous solution. The as-prepared solution was aged for about 10 hours. Before processing, the ITO substrates were thoroughly cleaned in acetone, ethanol, and deionized water of resistivity 18 MG for about 30 min inside an ultrasonic bath, and then dried under a stream of nitrogen. After that, the resulting solution was coated onto ITO-coated glass substrates at a fixed speed of 1000 rpm for 1 min with a spin coater at ambient temperature, as shown in Fig. 1. Subsequently, all samples were thermally treated on a hot plate in a protective atmosphere such as inert gas or reducing gas at an optimized temperature of 100 °C for 5 min to remove any undesirable organic compound. Lastly, the coating procedure cycle was done 5 times to realize the required thickness. The thickness of the spin-coated films ranges between 100 and 120 nm measured using a Bruker Dektak XT contact profilometer.

### 2.3 Characterization measurements

The binding energies (BE) of all the elements were performed using an ESCALAB 250 X-ray photoelectron spectrometer (XPS, Thermo Fisher Scientific). All the BE were calibrated to the C 1s peak of adventitious surface carbon at 284.8 eV. All chemical states of each element were determined according to the BE referenced in the National Institute of Standards and Technology (NIST) X-ray photoelectron spectroscopy database (NIST SRD 20) v. 5.0.^35^ The amount of Sb and S elements in all samples was measured by Inductively coupled plasma atomic emission spectroscopy (ICP-AES). Powder X-ray patterns were recorded on an automated Bruker D8 advance X-ray diffraction with a monochromatic CuK_α_ radiation source (*λ* = 1.5406 Å) in the angle range from 20° to 60°. The Xpert high-score software was employed for phase identification and structure refinement. Raman scattering spectroscopy was conducted on all synthesized samples using a Jobin Yvon LabRAM HR spectrometer with a He–Ne laser source (632.81 nm). The surface morphology was examined by using atomic force microscopy (AFM) (a Nanoscope 3100 Digital instrument in tapping mode). The grain size and root mean square (RMS) surface roughness were determined by using Gwyddion software version 2.43 developed by Czech Metrology.^36^ Hall effect measurement was conducted in Van der Pauw configuration with a constant magnetic field of 0.5 T to reveal the electrical properties (resistivity (*ρ*), carrier concentration (*N*_e_), and Hall mobility (*μ*_Hall_)) of obtained Sb_2_S_3_ thin films by using HMS5000 instrument. Optical transmission measurements were conducted by using NIR-UV-vis PerkinElmer Lambda 950 spectrophotometer equipped with an integrating sphere for light incidence in the wavelength range between 600 and 1200 nm at room temperature. Photoluminescence (PL) measurements were performed by using an Ar+ ion laser lamp emitting a wavelength of 266 nm at room temperature. The spectra were detected through a 250 mm Jobin-Yvon monochromator and a GaAs photomultiplier in conjunction with a standard lock-in technique. The surface wettability of Sb_2_S_3_ thin films was assessed through water drop contact angle (CA) measurements using the DSA100 drop shape analyzer system from the Kruss Easy Drop goniometer. Water drops with a volume of 10 μl were dispersed over the surface using a micro-syringe. Then, the CAs were obtained *via* the tangent technique using the DSA3 software incorporated with the device. Finally, the γ-irradiation was performed at the National Center for Nuclear Sciences and Technologies of Tunis with an industrial ^60^Co radioactive source with an irradiated dose of 0.25 kGy h^−1^. The obtained films were irradiated with γ-irradiation at different doses ranging from 5 to 20 kGy.

### 2.4 Photoelectrochemical characterizations

**Fig. 2:**
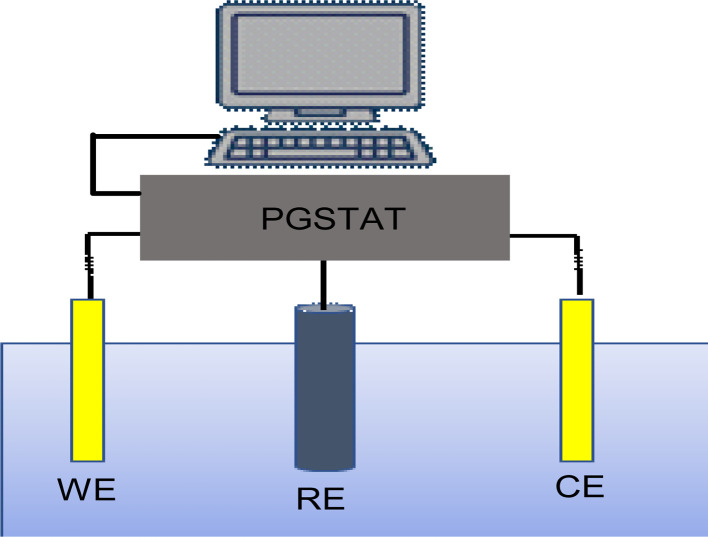
Illustration for the three-electrode setup used in the photoelectrochemical technique. WE = working electrode (Sb_2_S_3_ thin films); RE = reference electrode (SCE); CE = counter electrode (Pt plate).

The photocurrent was measured in a three-electrode system in the dark and under illumination on the synthesized samples with Sb_2_S_3_ thin films as the working electrode, saturated calomel as the reference electrode (SCE), and a Pt plate as the counter electrode. The data was achieved through a potentiostat/galvanostat Auto lab (model PGP201). These electrodes were vertically immersed in a 10 mL volume quartz cell, which contains Na_2_SO_4_ (0.5 M, pH ≅ 7) a buffer electrolyte solution, as shown in Fig. 2. Before the PEC measurements, the solution bath was degassed by bubbling with N_2_ gas for at least 10 min before commencing the experiment, and N_2_ was continuously bubbled through the solution using Sierra's flagship SmartTrak 100 thermal mass flow controller (flow rate = 5 mL min^−1^) throughout the measurement. Next, we apply a linear sweep voltammetry (LSV) mode with an anodic scan direction at the scan rate of 10 mV s^−1^ under chopped white illumination by a light-emitting diode (LED), which has been switched on and off with an asymmetric duty cycle. To enhance precision and reduce error estimation, our measurements were conducted five times under the same conditions. Mott–Schottky (M–S) measurements were carried out at an AC frequency of 1 kHz and an amplitude of 10 mV in dark conditions. All the measured potentials were converted to RHE scale using Nernst's equation:

1 *E* (V *vs.* RHE) = *E* (V *vs.* SCE) + 0.059 pH + 0.244

Incident photon-to-electron conversion efficiency (IPCE) and absorbed photon-to-current efficiency (APCE) measurements were collected at 0 V *vs.* RHE using a solar simulator coupled with a filter and an aligned monochromator in the same experimental setup as described above. IPCE and APCE values are determined using the following equation:2
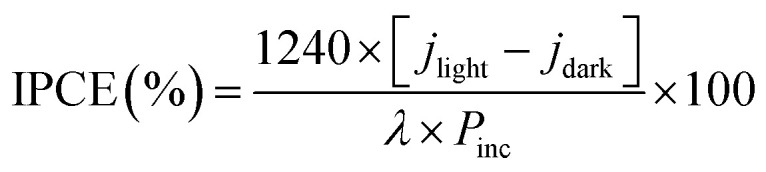
3
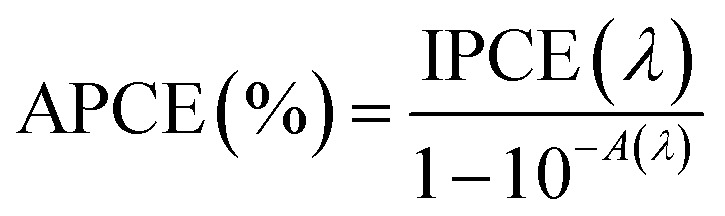
where *j*_light_ is the steady-state photocurrent density, *j*_dark_ is the dark current density (mA cm^−2^), *P*_inc_ is the incident optical power density of monochromatic light (mW cm^−2^), *λ* is the monochromatic wavelength of light (nm), and *A* (*λ*) is the optical absorption of the entire photoelectrode.

Applied bias photon-to-current efficiency (ABPE) for the PEC cell was calculated using the following relationship:4
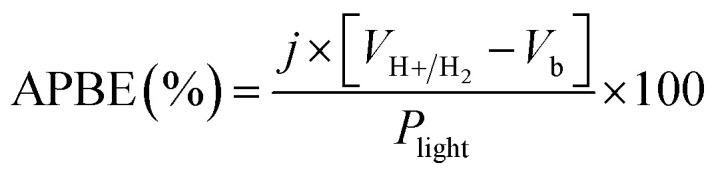
where *V*_b_ denotes applied bias voltage *vs.* RHE, *V*_H+/H_2__ is the thermodynamic potential for hydrogen evolution, and *J* is the measured photocurrent current density (mA cm^−2^) taken after 30 s illumination at each *V*_b_. *P*_light_ is the incident light power density (100 mW cm^−2^).

## 3. Results and discussion

### 3.1 XPS and ICP-AES analysis

**Fig. 3:**
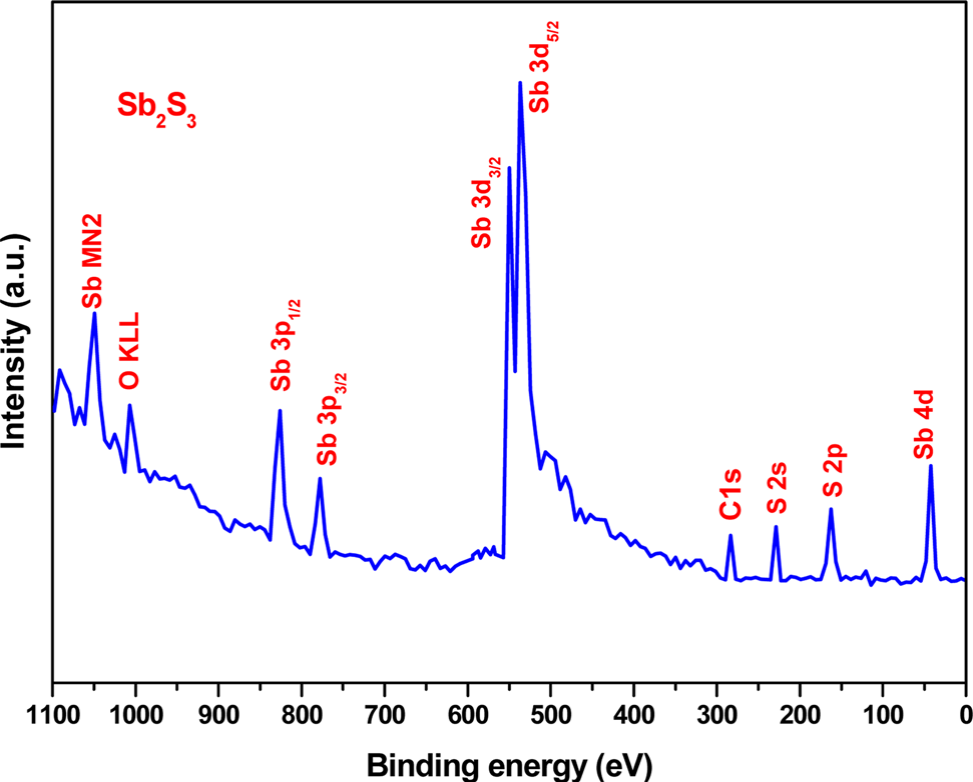
High-resolution XPS spectra of bare Sb_2_S_3_ film.

**Fig. 4:**
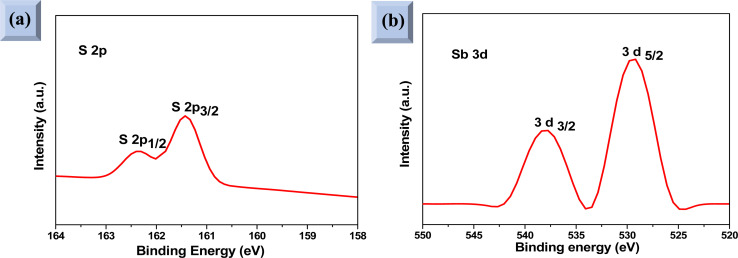
High-resolution XPS spectra of (a) S 2p and (b) Sb 3d core levels of the bare Sb_2_S_3_ material.

**Fig. 5:**
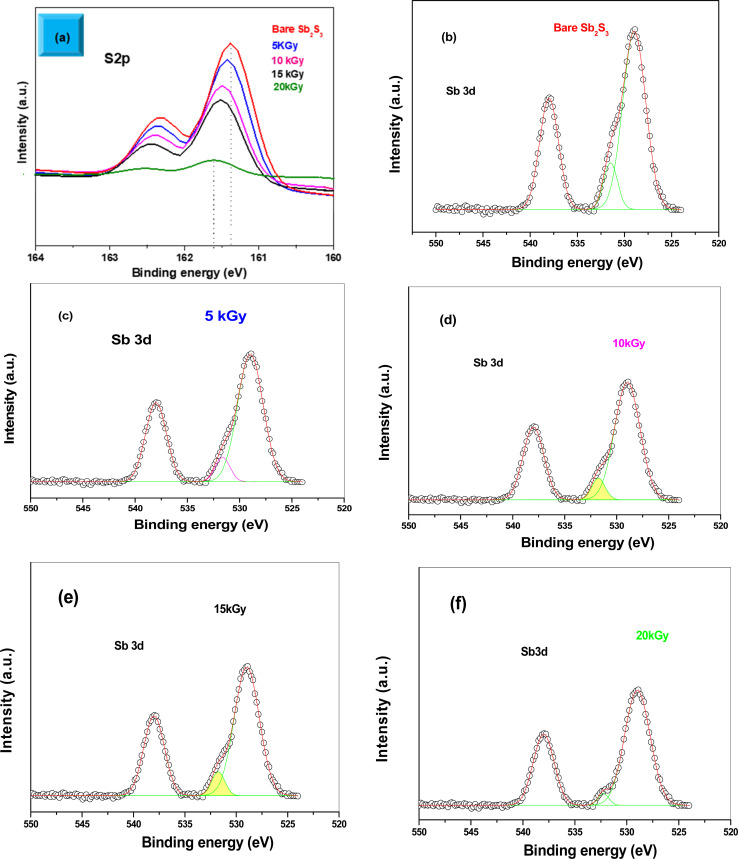
High-resolution XPS spectra of S 2p with various γ radiation doses (a). High-resolution XPS spectra of Sb 3d with various γ radiation doses (b–f).

XPS analysis was performed to assess whether any alterations in the elemental composition and chemical states of Sb_2_S_3_ thin films occurred following γ-ray irradiations. Fig. 3 shows the XPS survey spectrum of the bare Sb_2_S_3_ film, covering a BE range from 0 to 1100 eV. The BE photoelectron lines of all XPS spectra are consistent with previous research studies.^22^ The photoelectron lines of oxygen and carbon elements are likely attributed to the formation of reactive oxide species and surface impurities, which are common in chalcogenide films when they encounter atmospheric air.^37^ The presence of S^2−^ ions in the bare Sb_2_S_3_ material is indicated by the photoelectron lines for S at approximately 161.4 eV and 162.3 eV, which are assigned to the doublet S 2p_3/2_ and S 2p_1/2_, respectively,^38^ as shown in Fig. 4a. The corresponding spin–orbit splitting of S 2p is about 0.9 eV, while the intensity ratio of the two photoelectron lines in the doublet is S 2p_3/2_ : S 2p_1/2_ = 2 : 1. On the other hand, Sb photoelectron lines showed BE of 529.8 eV and 539.0 eV approving the presence of Sb 3d_5/2_ and Sb 3d_3/2_ states, respectively. The matching spin–orbit splitting of the Sb 3d photoelectron line is about 9.2 eV, while the two relative line intensities in the doublet are Sb 3pd_5/2_ : Sb 3p_3/2_ = 3 : 2, which suggests the existence of Sb^3+^ valence states,^39^ as shown in Fig. 4b. No satellite photoelectron lines were detected indicating that Sb is present only as Sb^3+^ ions. Considering the neutrality of the material, the XPS results suggest that the valence states for our synthesized Sb_2_S_3_ material are Sb_2_^3+^S_3_^2−^. To further investigate the influence of γ-ray irradiations on the chemical states of Sb_2_S_3_ thin films, the XPS spectrum of S and Sb with different irradiation doses is revealed in Fig. 5. It is widely recognized that XPS is primarily sensitive to the surface composition of the film, typically examining only the top 10 nm.^40^ Thus, we anticipate that there will be no significant variation in the chemical composition of the Sb_2_S_3_ film throughout its thickness after γ-ray irradiation. The spectral shape of both S and Sb core level spectra in bare and irradiated samples is nearly identical. This implies that in the irradiated Sb_2_S_3_ samples, the radiation dose does not promote the creation of an additional phase. It is interesting to note that the irradiated Sb_2_S_3_ samples showed an upward shift to the left (*i.e.*, higher BE) of the S 2p core level photoelectron lines by about 0.18 eV compared to bare Sb_2_S_3_, indicating a slight change in the chemical environment of the sulfur atoms, as shown in Fig. 5a. However, the photoelectron line separation remained unaffected at 0.9 eV, meaning that the valence state of the S ion was still S^2−^. Conversely, the signal intensity of S sites in the irradiated samples appears to decrease as the radiation dose increases. Beyond a radiation dose of 15 kGy, the doublet S 2p photoelectron lines exhibit a decrease in intensity of approximately 4-fold compared to their corresponding photoelectron lines intensities in the bare Sb_2_S_3_ sample. The observation that the photoelectron line shows an intensity change over 15 kGy indicates a significant influence on the electronic structure of Sb_2_S_3_ films. This threshold behavior highlights the need for careful consideration of irradiation doses in understanding and controlling the electronic properties of the Sb_2_S_3_ material. We speculate two main factors that contribute to this behavior: (i) the decrease in the number of sulfur sites on the Sb_2_S_3_ surface with increasing γ irradiation; and (ii) the presence of an ionization process in the experimental setup.^41^ Consequently, the radiation could induce an increase of S vacancies in the Sb_2_S_3_ structure. Likewise, the XPS spectrum of the Sb 3d_5/2_ photoelectron line shows a broader peak before the irradiation, which decreases in intensity after the irradiation without any significant shifting in their BE positions, as illustrated in Fig. 5(b)–(f). The observed sharpening of the Sb 3d_5/2_ peaks is attributed to a reduction in the oxygen content in the Sb_2_S_3_ samples after γ irradiation. A common problem when analyzing Sb-XPS data is the possibility of overlap between the photoelectron lines of Sb 3d and O 1s.^42^ To resolve overlapping photoelectron lines in the Sb-XPS survey spectrum, we employed the deconvolution process using Origin software to determine the various elemental concentrations. Through spectral analysis of the Sb-XPS data, we found that fitting the photoelectron lines using Voigt functions provides a more accurate representation of the oxidation states. In the case of a bare sample, some of the O 1s and Sb 3d_5/2_ photoelectron lines typically merge and form a broader width peak. However, the loss of oxygen from the Sb_2_S_3_ samples during γ irradiation leads to a decrease of full width at half maximum (FWHM), as shown in Table 1. This result exhibits a reduction of Sb 3d_5/2_ photoelectron lines, potentially attributed to the decrease in oxygen vacancies. On the other hand, the surface atomic ratio of Sb/O is calculated following this formula Sb/O = 
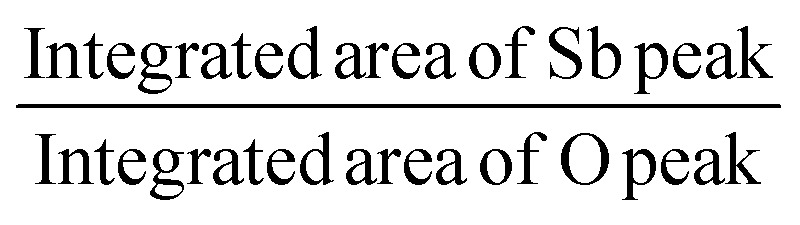
 in the XPS spectra. As can be seen, the surface atomic ratio of Sb/O exhibited a gradual increase with higher radiation doses. The atomic ratio of Sb/O estimated from the surface area in the bare sample is approximately 1.21, which is significantly lower than the sample exposed to a radiation dose of 20 kGy, where the atomic ratio is 1.35. This can be attributed to the annihilation of microcrystalline Sb oxide species formed on the surface at these elevated doses. A similar result has been reported for Y_1_Ba_2_Cu_3_O_7−*δ*_ superconductors, where γ radiation was used to decrease the oxygen level.^43^ Moreover, there is a decreasing trend in the intensity of the Sb 3d_5/2_ photoelectron line following γ irradiation. In comparison to a bare sample, the intensity of this photoelectron line is significantly decreased by a factor of five when exposed to an irradiation dose of 15 kGy. These alterations in photoelectron line intensity are likely the result of changes in element levels induced by γ irradiation. Such changes could be ascribed to bond breaking and subsequent reorganization in the Sb_2_S_3_ samples. It is also plausible that the variation in element contents in the Sb_2_S_3_ sample is due to the diffusion of ions from the surface to the bulk material and *vice versa*, as well as the decrease in oxygen content after γ irradiation. In this regard, the likelihood of a change in Sb content due to a change in the oxidation state from Sb^5+^ to Sb^3+^ is excluded since such changes are not stable and recover quickly.^44^ Overall, the results of the XPS measurements reveal the formation of S vacancies and loss of oxygen content in the irradiated samples. Thus, the charge carrier separation could have occurred in the irradiated samples. Our results are consistent with those of Wang *et al.* who found that S vacancies introduced by γ-irradiation on the Zn side can accelerate the separation of charge carriers by trapping electrons that are transferred to the catalyst surface.^27^ In line with XPS analysis, ICP-AES further investigates the atomic proportions of bare and irradiated samples, as shown in Table 2. By comparing the atomic proportions of all samples, the ICP-AES result is consistent with the results of the XPS analysis and confirms the accuracy of the elemental composition of the material.

**Table 1:** FWHM of 3d_5/2_ photoelectron line as a function of gamma irradiation dose

Gamma irradiation dose (kGy)	FWHM (eV)
0	2.20
5	2.16
10	2.12
15	1.90
20	2.15

**Table 2:** Composition of different films from ICP-AES data

Gamma irradiation dose (kGy)	Sb (at%)	S (at%)	Total
0	38.92	61.08	100
5	39.15	60.85	100
10	39.46	60.54	100
15	40.80	59.20	100
20	38.41	61.59	100

### 3.2 Structural properties

**Fig. 6:**
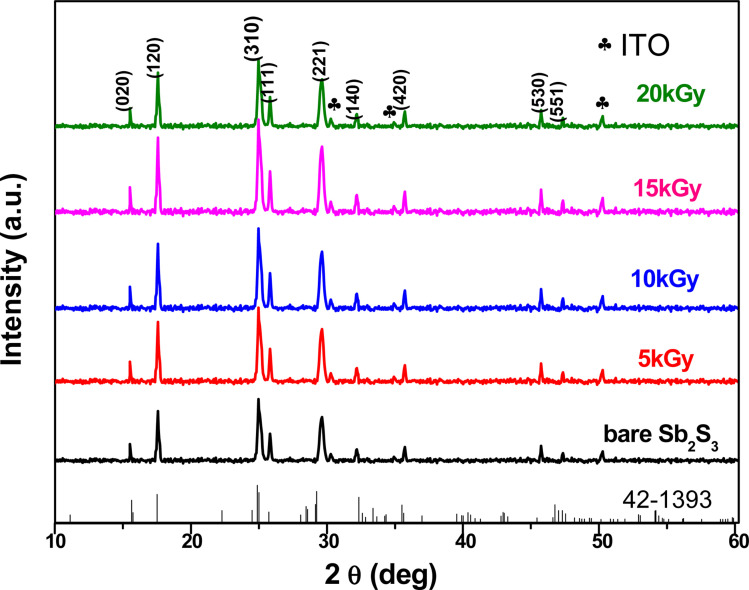
XRD pattern of Sb_2_S_3_ before and after irradiation with various γ radiation doses.

**Fig. 7:**
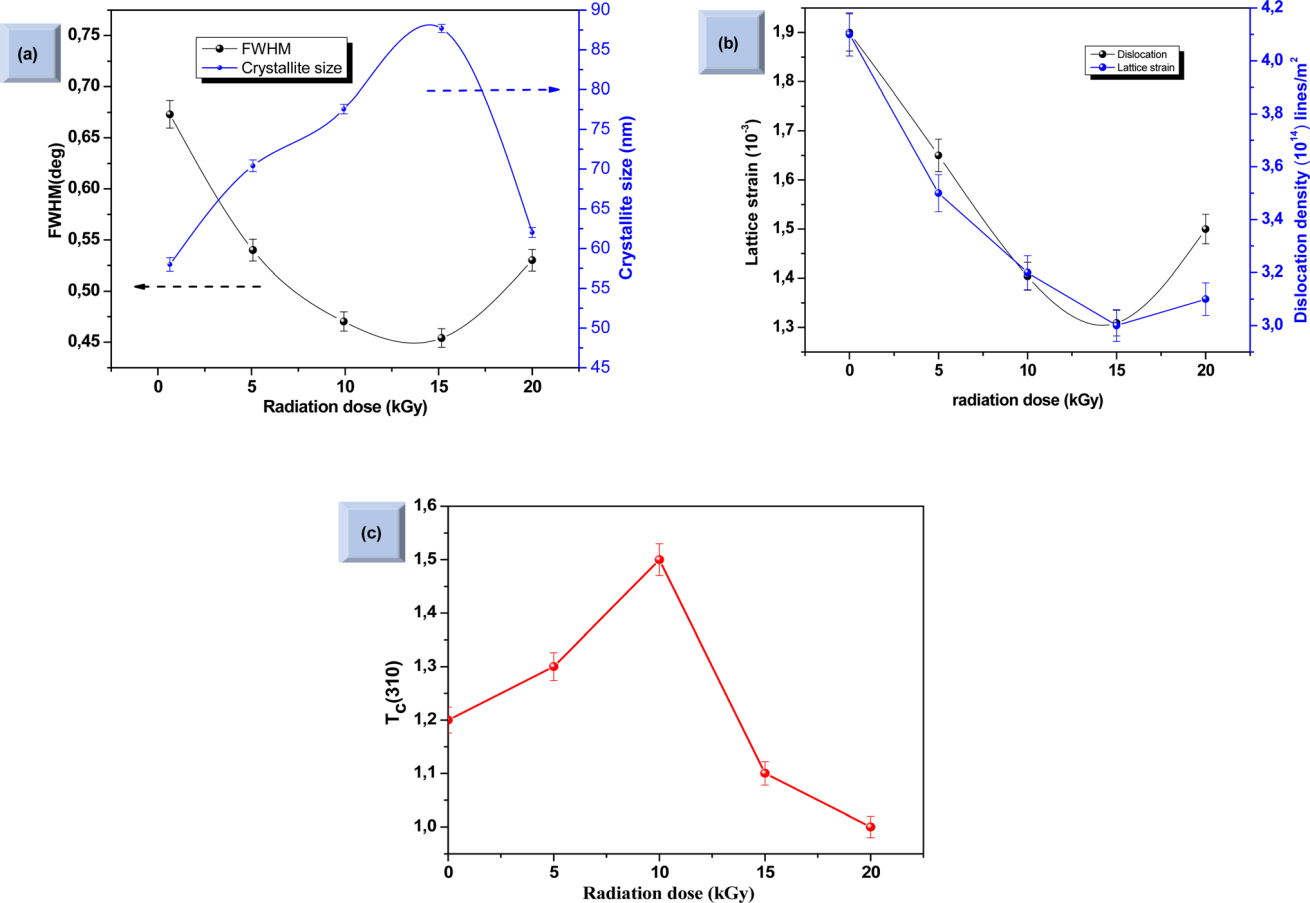
Variation of full width at half maximum (FWHM) and crystallite size as a function of irradiation doses (a). Variation of dislocation density (*δ*) and lattice strain at different irradiation doses (b). Influence of γ radiation on texture coefficient *T*_c_ (310) (c). Each data point in the two graphs is accompanied by error bars that represent a 2% margin of uncertainty and provide a quantifiable measure of the variability in our measurements.

The crystalline structure and phase purity of the bare and irradiated Sb_2_S_3_ thin films were characterized by X-ray diffraction (XRD) from 2*θ* = 10° to 2*θ* = 60°. Fig. 6 shows the XRD patterns of bare and irradiated thin films exposed to various γ ray energies. XRD peaks of the samples are in good agreement with the orthorhombic crystal structure of Sb_2_S_3_ (stibnite)^45^ with the lattice constants *a* = 11.310 Å, *b* = 3.836 Å, *c* = 11.228 Å and *Pbnm* space group symmetry, which is in line with the literature values of the standard JCPDS card no: 42-1393. What is more, there are no impurities or unwanted phases present in the XRD patterns as the γ ray energies increase. This indicates that the material's crystalline structure remains relatively stable under the influence of gamma radiation. The other diffraction peaks indexed by hashtags stand for ITO glass substrate (JCPDS 06-0416). It is worth noting that the intensity of the prominent peaks located at 2*θ* = 17,52°, 2*θ* = 24,88°, and 2*θ* = 29,24° gradually increases with increasing gamma irradiation, and the FWHM of the diffraction peaks decreases up to irradiation dose of 15 kGy. However, above this value, the peak intensity decreases and the FWHM increases slightly due to the structural disorder created after the irradiation process. The initial intensification and the sharpening of the peak intensity with γ-ray irradiations can be attributed to an enhancement in crystallinity. However, the reduction is attributed to the impact of ionizing γ-ray irradiations, allowing Sb and S atoms to be shifted and the molecules to be broken down. A similar effect has previously been described for WO_3_ thin films grown by RF sputtering.^46^ This suggests that the gamma irradiation process might have positively influenced the charge carrier dynamics within the Sb_2_S_3_ material. Understanding and harnessing such effects could have significant implications for the development of efficient photocatalysts for PEC cells. Our findings are somewhat surprising since γ-ray radiations are supposed to generate structural defects, disordering and clustering, swelling, and polygonization leading to a drop in peak intensity.^47^ Nevertheless, γ-ray irradiations could induce a self-annealing process in the Sb_2_S_3_ films by increasing the kinetic energy of atoms, subsequently an improvement in crystal quality. These observations agree well with previously reported results for irradiated semiconductors.^48^ The enhanced crystal structure leads to higher charge transfer mobility and light absorption.^49^ In this regard, γ ray energy could improve the crystallinity of the Sb_2_S_3_ films and promote its PEC properties. It is also worthwhile to note that the orthorhombic crystal structure and diffraction angles of Sb_2_S_3_ films did not change after γ-ray irradiation. This implies that Sb_2_S_3_ films have structural integrity even after γ-ray irradiations. Such results indicate that Sb_2_S_3_ has notable bonding strength and high γ-ray hardness. Other semiconductor materials that have been significantly spoiled by γ irradiation have not demonstrated such resilience.^50^ To further investigate the XRD measurements, the FWHM of the dominant peak (310) and the average crystallite size *versus* of γ irradiation dose was calculated for all the samples. Note that the FWHM of the peak was found by fitting the XRD patterns using Origin software. The data on the structural characteristics of the Sb_2_S_3_ thin films were estimated by computing the average crystallite size (*D*), the lattice strain (*ε*), as well as the dislocation density (*δ*) according to the following equations:5
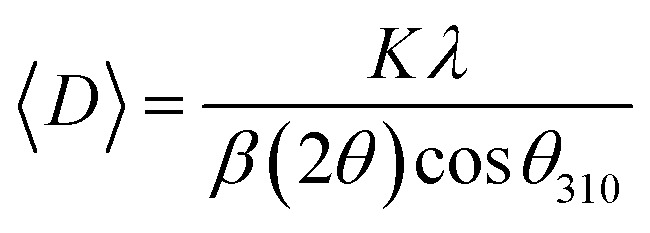
6
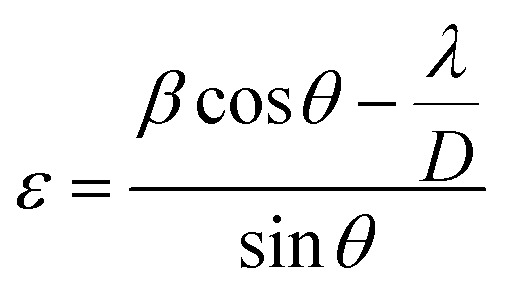
7
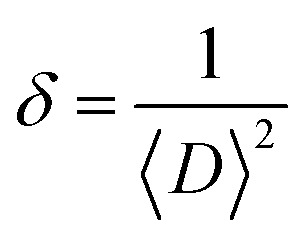
where *λ* is the wavelength of incident X-ray radiation (1.5406 Å of CuK_α_), *β* is FWHM, *θ* is the Bragg's angle in radians, and *K* can have values anywhere from 0.62 and 2.08. In this paper, *K* = 0.9 was used. As shown in Fig. 7a, increasing the γ irradiation dose results in a decrease in FWHM from 0.67° to 0.45° when the irradiation dose varies between 0 and 15 kGy. The average crystallite size reaches its maximum value of 87.4 nm at a γ radiation dose of 15 kGy. Conversely, with an increase in γ-radiation dose up to 15 kGy, the dislocation density and lattice strain of Sb_2_S_3_ thin films decrease. Subsequently, beyond 15 kGy, they increase, as shown in Fig. 7b. The increase in crystallite size and reduction in GBs, lattice strain, and dislocation density with the increase in γ-radiation dose suggest improvements in crystal lattice quality. This is often accompanied by a more orderly and error-free structure of the Sb_2_S_3_ thin films.

**Fig. 8:**
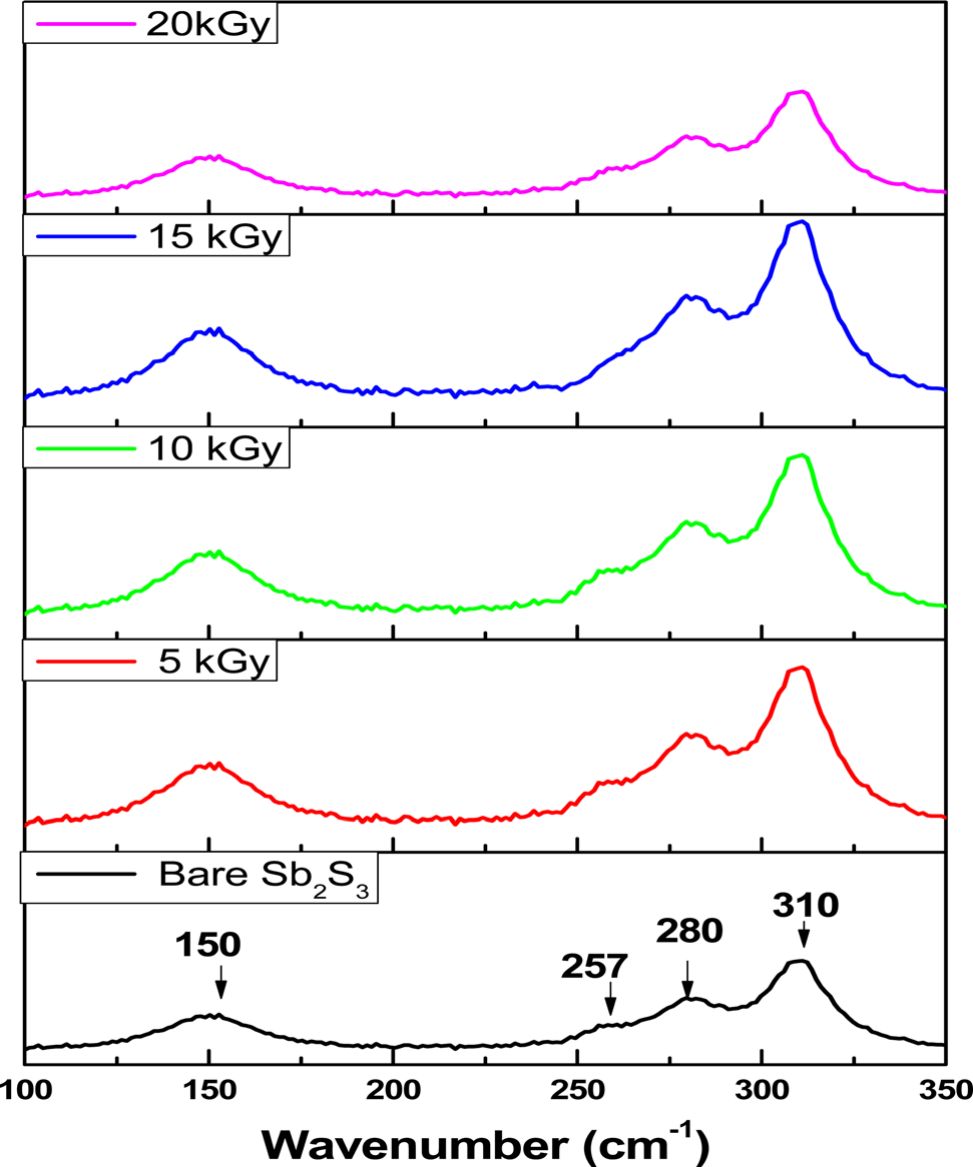
Typical micro-Raman spectra before and after irradiation with different irradiation doses.

The texture coefficient *T*_c_(*hkl*) is a parameter used to quantify the preferred orientation of a plane (*hkl*) and is estimated using the following equation:8
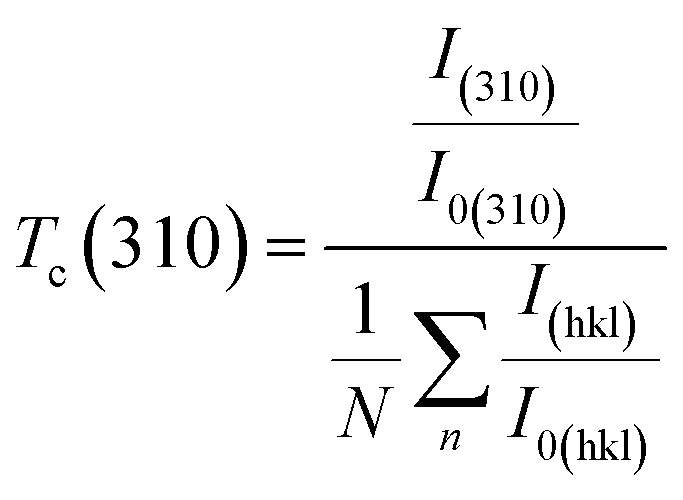
where *I*_(310)_ and *I*_(*hkl*)_ are the intensities of the (310) crystal plane and a general peak with Miller indices (*hkl*), respectively. *I*_0(310)_ and *I*_0(*hkl*)_ are the integrated intensities of the JCPDS (powder diffraction pattern) data of the corresponding plane (310) and (*hkl*) of the Sb_2_S_3_ material, respectively. *N* is the number of reflection peaks, and *n* = 9 is the number of diffraction peaks measured in the analysis. When *T*_c_ (310) = 1 that means no preferred orientation (*i.e.*, the crystallites are randomly oriented), while a *T*_c_ (310) > 1 indicates the preferred orientation of the crystallites is in (310) direction. A *T*_c_ (310) < 1 means that the [310] orientation is less preferred than in a randomly oriented sample. Fig. 7c shows that most films have a *T*_c_ > 1, indicating the presence of preferred (310)-oriented crystallites that increase with γ radiation compared to the bare Sb_2_S_3_ sample. These results indicate that γ radiation can confirm the Sb_2_S_3_ crystal orientation of the preferred [310] orientation. Such preferential orientation was previously observed for Sb_2_S_3_ thin films deposited using vapor transport deposition methods,^51^ and solvothermal method.^52^ To further scrutinize the phase purity of Sb_2_S_3_ material, Raman measurement was performed on the deposited thin films at room temperature. Fig. 8 depicts the Raman spectra collected before and after irradiations between wavenumbers of 100–350 cm^−1^ at different radiation doses. The bands located at 150, 280, and 310 cm^−1^ can be assigned to specific vibrational modes related to the orthorhombic Sb_2_S_3_ phase corresponding to Sb–Sb bond, antisymmetric Sb–S stretch, and symmetric Sb–S stretch in orthorhombic Sb_2_S_3_ phase, respectively.^53^ The bands located at 280 cm^−1^ and 310 cm^−1^ were attributed to the symmetric vibrations of a *C*3v symmetric cone element, as reported in previous studies.^54^ The spectrum of the bare sample reveals also a weak band located around 257 cm^−1^ suggesting the presence of Sb_2_O_3_,^55^ which corroborates the XPS results.

The weak band at 257 cm^−1^ reduces with increasing gamma radiation exposure; however, it never completely disappears due to the residual Sb_2_O_3_ phase present in the synthesized material. Thus, when the surface defects are created owing to γ irradiation, the oxygen present in the environment interacts with these defects leading to the formation of Sb_2_O_3_. Nevertheless, the existence of the Sb_2_O_3_ phases could not be observed in the XRD pattern. This discrepancy between XRD and Raman results would be due to chemical sensitivity. XRD can give information about the arrangement of atoms, but it might not distinguish between different chemical species that have similar crystal structures. It is worth noting that no bands belonging to other foreign phases or impurities such as S_8_, and SbS_3_ were detected, suggesting moderately high purity of the formed Sb_2_S_3_ thin film. On the other hand, the band intensity of the Raman spectra increased significantly when the irradiation dose increased up to 15 kGy without any shift. This implies that ionizing energy might strengthen the Sb_2_S_3_ orthorhombic crystal structure and improve its quality, which is in line with the XRD results. In addition, the Sb_2_O_3_ phase gradually vanishes, signifying a purer fabricated Sb_2_S_3_ film. As a result, Sb_2_S_3_ thin films after the ionizing radiation process are almost free of Sb_2_O_3_ phases with a sulfur vacancy.

### 3.3 Scanning electron microscope (SEM) measurements

**Fig. 9:**
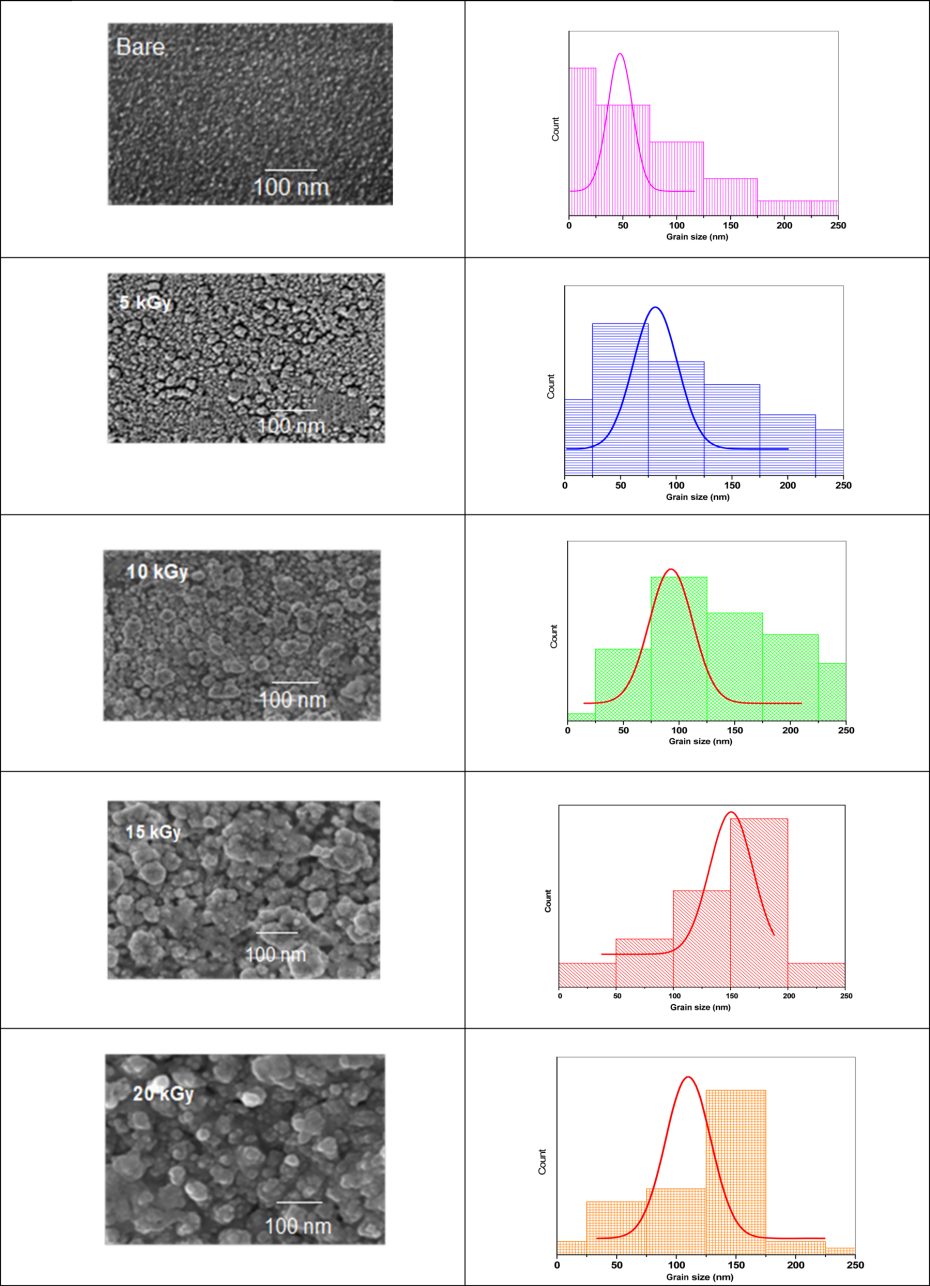
SEM images of Sb_2_S_3_ thin films before and after irradiation with different irradiation doses.

To unearth the impact of γ-irradiation on the surface morphology of the synthesized photoelectrodes, we conducted SEM measurements on Sb_2_S_3_ films and analyzed the quantification of grain sizes. As depicted in Fig. 9, the impact of γ-irradiation below 15 kGy conspicuously increases the grain size. Meanwhile, the compactness and surface smoothness of film were boosted greatly with increasing γ-irradiation doses. The corresponding histograms illustrating the size distribution of the Sb_2_S_3_ grains are presented in Fig. 9. For the film irradiated with 15 kGy, it demonstrates an average size of about 151 nm. However, beyond this dose, the grain size of the film decreases and its compactness deteriorates. It is also worthwhile to note that the voids and shape irregularities observed in the as-deposited thin film have been significantly reduced compared to irradiated films. They are substituted by more regular grain shapes, resulting in a reduction in GBs. This observation is consistent with the enhancement of crystallinity in the irradiated thin film, as described earlier through XRD analysis.

### 3.4 Atomic force microscopy (AFM) measurements

**Fig. 10:**
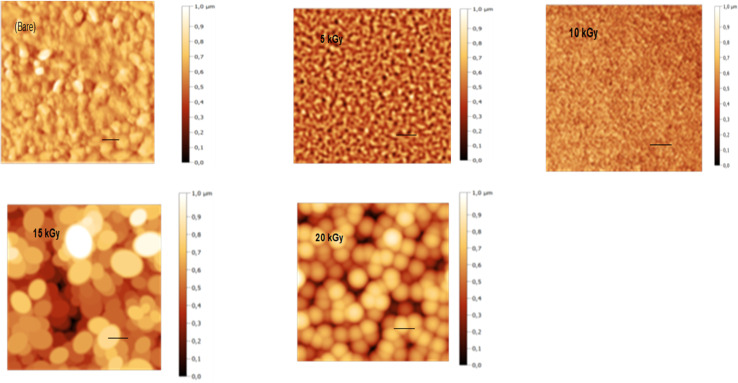
2D AFM micrographs of bare and irradiated Sb_2_S_3_ thin films at different doses. The scale bar on all images indicates a length of 100 nm.

AFM with a contact mode was utilized to depict the 2D morphology of all Sb_2_S_3_ samples grown on an ITO substrate with a scanning area of 1 μm × 1 μm under various γ-irradiation doses ranging from 0 to 20 kGy. As depicted in Fig. 10(a)–(e), the morphology of the examined films is significantly influenced by the γ-irradiation. Fig. 10a shows the morphology of the bare Sb_2_S_3_ film with random grains and spherical shapes, but not uniformly distributed across the entire surface. Nevertheless, as the dose increases, the topography of the Sb_2_S_3_ film changes, and more spherical particles become evident, as displayed in Fig. 10(b)–(e). Indeed, γ-irradiation can induce changes in the material properties, such as compaction or densification. This can cause the spherical shapes to become more compact and spherical in appearance when imaged with AFM. The average grain size of Sb_2_S_3_ films gradually increased with γ-irradiation doses and reached its maximum at 180 nm, as shown in Table 3. The grain sizes estimated by AFM measurements are somewhat larger than those determined by the Scherrer equation. The overestimated grain size values can be attributed to the fact that AFM measurements directly capture the surface morphology of agglomerated grains and thus provide information about the particle size. The obtained average grain size can improve the optical scattering by increasing the absorption coefficient, and this can also affect the film resistivity. On the other hand, the RMS of the surface roughness *R*_q_ is an important parameter that provides a measure of the average magnitude of surface deviations from the mean plane^56^ and is computed using the following equation:9
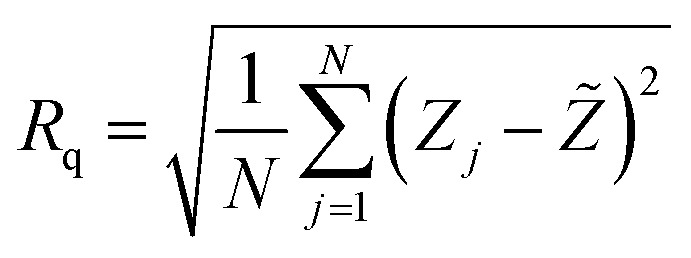
where *N*, *Z*_*j*_, and *Z̃* are the number of points within the given area, the height deviation of each data point, and the mean height, respectively. Table 3 exhibits the variation of *R*_q_*versus* the γ-radiation dose. The RMS surface roughness increases progressively and reaches its maximum value of about 38 nm for the irradiated sample with 15 kGy film. With an increase in gamma irradiation at a dose of 20 kGy, the *R*_q_ value decreases to about 30 nm because more defects and sulfur vacancies appear on the surface. From the obtained AFM results of Sb_2_S_3_ samples, the variation of *R*_q_ is consistent with the variation of crystallite size from XRD analysis. It is established that an increase in surface roughness results in a higher number of active sites on the surface, and the GBs become smaller. This morphology is beneficial for PEC performance^57^ since it suppresses the recombination of excitonic pairs. A rougher film surface is expected to have a larger electrode/electrolyte interface area, thereby increasing the rate of charge transport relative to recombination.

**Table 3:** RMS surface roughness as a function of gamma irradiation dose

Gamma irradiation dose (kGy)	RMS surface roughness	Average grain size of Sb_2_S_3_ (nm)
0	15	124
5	21	145
10	29	168
15	38	180
20	30	171

### 3.5 Hall effect measurement

**Fig. 11:**
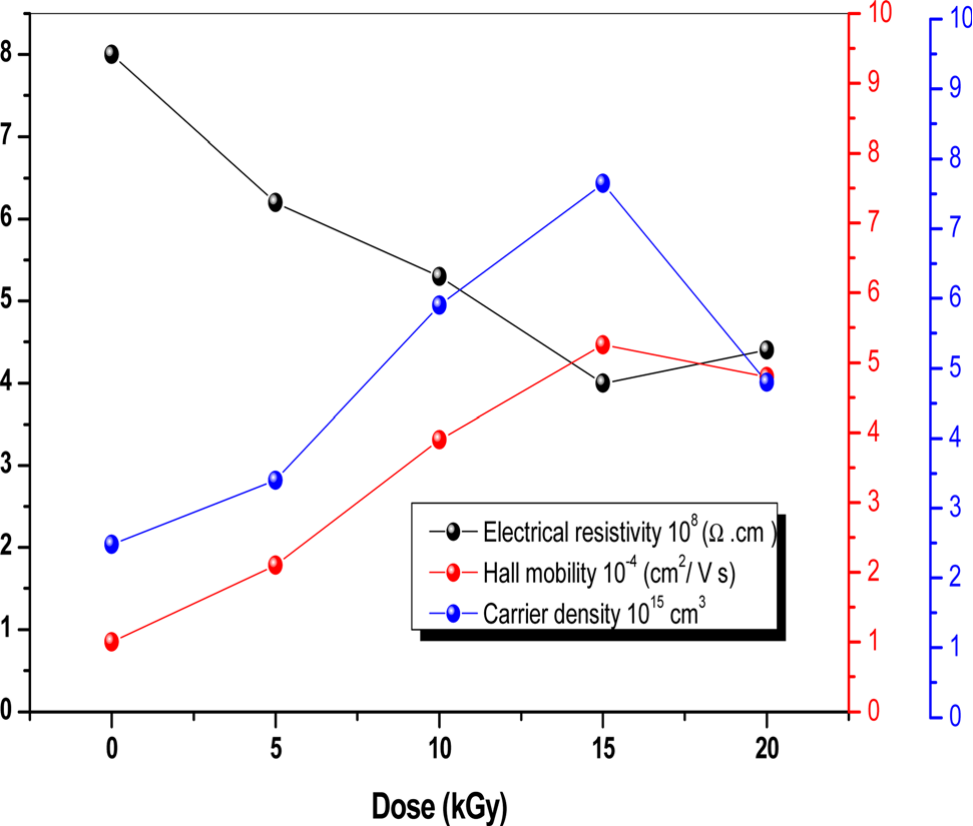
Electrical resistivity, Hall mobility, and carrier density of Sb_2_S_3_ thin films before and after γ-irradiation with different doses.

Hall effect measurement is a straightforward method for determining majority carrier density, electrical conductivity, and Hall mobility (*μ*_HALL_) in semiconductor materials. It was used for both bare and irradiated Sb_2_S_3_ films synthesized on the ITO substrate under consistent conditions including a constant electric current of 10 μA and a constant magnetic field of about 0.5 T using Van der Pauw geometry at room temperature. It is interesting to note that gamma irradiation does not seem to have a discernible impact on the electrical conductivity type of the Sb_2_S_3_ films, which consistently exhibit p-type behavior across different irradiation doses. Thus, there is no transition from p-type to n-type conductivity under gamma irradiation. However, it is worth noting that gamma irradiation may impact other parameters of Hall effect measurements charge carrier mobility (*μ*_H_), charge density (*N*), Hall coefficient (*R*_H_), and resistivity (*ρ*). The p-type conductivity observed in the obtained Sb_2_S_3_ films is attributed to sulfur loss, as discussed earlier in the XPS section. This correlation between the sulfur loss and the resulting p-type conductivity aligns with previously reported research on Sb_2_S_3_ thin films.^58^ It is worth noting that p-type conductivity for Sb_2_S_3_ is often reported in the literature, whereas n-type behavior is rarely reported.^17^ According to the first principles of density functional theory (DFT), the Sb_2_S_3_ films exhibit p-type semiconductor character due to S-on-Sb antisite (SSb) and to the S-vacancies.^59^ While Rajpure *et al.*^60^ showed that the electrical conductivity in Sb_2_S_3_ switches from n-type to p-type only by modifying the precursor solvent from an aqueous to a non-aqueous solution. Fig. 11 illustrates the effect of γ irradiation on the electrical resistivity (*ρ*), hole density (*N*_h_), and hole mobility (*μ*_HALL_). The electrical resistivity tends to decrease *versus* γ irradiation dose in the range of 0–15 kGy. When the γ irradiation doses increase, the electrical resistivity declines gradually from 8 × 10^5^ Ω cm (0 kGy) to 4 × 10^5^ Ω cm (15 kGy), which means the electrical conductivity increases. The obtained results can be attributed to the improved crystalline quality and the lack of trap states (S_Sb_ and V_Sb_), providing support for the observed increase in the conductivity of the Sb_2_S_3_ layers. Recent studies have demonstrated that the electrical properties of Sb_2_S_3_-based thin films are significantly influenced by the crystallographic orientation of the polycrystalline domains.^61^ This suggests an intrinsic connection between the electrical properties of these materials and their crystalline structures and morphologies. Normal grain growth was observed when the grains were vertically stacked on the bonded substrates through effective covalent Sb–S bonds, resulting in [*hk*1]-oriented films, which are useful for charge carrier transport along the perpendicular direction.^62^ In contrast, γ irradiation promoted abnormal [Sb_4_S_6_]_*n*_ ribbons grain growth with parallel orientation to the substrates through weak van der Waals forces, thereby favoring [*hk*0]-oriented Sb_2_S_3_ film growth. Hence, it can be inferred that the Sb_2_S_3_ thin film with the preferred [310] orientation enables higher conductivity and more efficient separation of a photogenerated carrier in the corresponding PEC device compared to the typically [*hk*1]-oriented films. According to Vadapoo *et al.* the conductivity of Sb_2_S_3_ film at room temperature is 100-fold smaller along the perpendicular (Sb_4_S_6_)_*n*_ ribbon direction than in the parallel direction.^63^ Therefore, it is believed that gamma irradiation could induce a preferential crystallographic direction and improve the conductivity of Sb_2_S_3_ films more efficiently. On the other hand, with increasing irradiation dose up to 15 kGy, *μ*_HALL_ consistently raises from 10^−4^ cm^2^ V^−1^ s^−1^ to 5.25 10^−4^ cm^2^ V^−1^ s^−1^, and the hole density raises from 2.48 × 10^15^ cm^3^ to 7.65 × 10^15^ cm^3^. This increase in carrier mobility and hole density could be related to an improvement in microstructure and morphology as shown by AFM studies. Nevertheless, above a dose of 15 kGy, the electrical resistivity increases, the electron density decreases, and *μ*_HALL_ drops to a value of about 3.75 × 10^−4^ cm^2^ V^−1^ s^−1^. This could be described by the fact that excessive γ-irradiation may generate too many lattice defects, such as dislocations and/or GBs acting as electron–hole recombination centers.^64^ Finally, our results suggest that irradiation is a promising method for maintaining electrical conductivity type, however, its variability at various doses could be employed as a potential technique for detecting ionizing radiation.

### 3.6 Optical absorption properties

#### 3.6.1 UV-VIS spectroscopic measurements

**Fig. 12:**
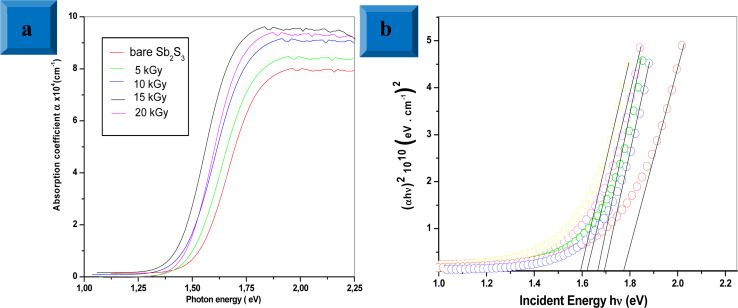
UV-vis absorption spectra for bare and irradiated Sb_2_S_3_ thin films at different doses (a). Tauc plot for bare and irradiated samples was obtained from the absorption spectra; the solid lines represent the linear regressions on the data from which the optical band gap energies were determined (b).

Fig. 12a exhibits the optical absorption spectra of bare and irradiated Sb_2_S_3_ films in the photon energy range of 1 eV to 2.25 eV. The linear optical absorption coefficient *α*(*λ*) was estimated from the transmittance and the reflectance spectra in the strong absorption region and achieved by the well-known equation:10
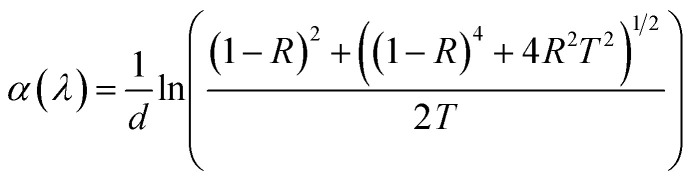
where *d* is the thickness of the deposited films, *R* and *T* are the reflectance and transmittance, respectively. The results show that the average absorption coefficient gradually increases with increasing irradiation doses. Especially, the irradiated 15 kGy Sb_2_S_3_ film exhibits a higher absorption coefficient. This increase could be attributed to the enhancement of crystallinity as discussed above in the XRD section. On the other hand, the optical band gap energy for all the Sb_2_S_3_ films is estimated from Tauc plot using the following equation:

11 (*αhν*) = *A*(*hν* − *E*_g_)^*n*^

where *α*, *A*, *h*, *ν*, *E*_g_ and *n* stand for absorption coefficient, a constant parameter, Planck's constant, the frequency of incident light, the optical band gap energy, and *n* constant linked to the type of optical transition (*n* = 2 for indirect band gap and *n* = 1/2 for direct band gap), respectively. So, we plot the variation of (*α h ν*) as a function of (*h ν*)^1/2^, then we fit the linear portion of the curve and extend it to the *x*-axis for all synthesized samples. Upon γ irradiation, the optical band gap is found to gradually decrease from 1.78 eV to 1.60 eV until radiation dose of 15 kGy and marginally increases at 1.64 eV, thereafter, as shown in Fig. 12b. The initial red shift in the optical band gap value suggests that gamma irradiation introduces additional electrons, causing the Fermi level to rise. The rise in the Fermi level results in the formation of impurity energy levels at the bottom of the conduction band or valence band, potentially leading to enhanced catalytic activity upon photoexcitation. Similar effects have been reported by other studies.^65^ Considering the forecast band gap of 15 kGy Sb_2_S_3_ film, it is a prone candidate for use in a photoelectrochemical cell. It is well known that a single semiconductor with a bandgap of 1.60 eV can achieve a maximum STH efficiency of 30% at one solar irradiance.^66^ However, for a higher irradiation dose above 15 kGy, the broadening of the band gap could be explained by the energy width of band tails of the localized state. Therefore, further irradiation dose by over 15 kGy could not decrease the band gap any further but controversy the band gap increases at 1.64 eV.

#### 3.6.2 Photoluminescence spectroscopy

**Fig. 13:**
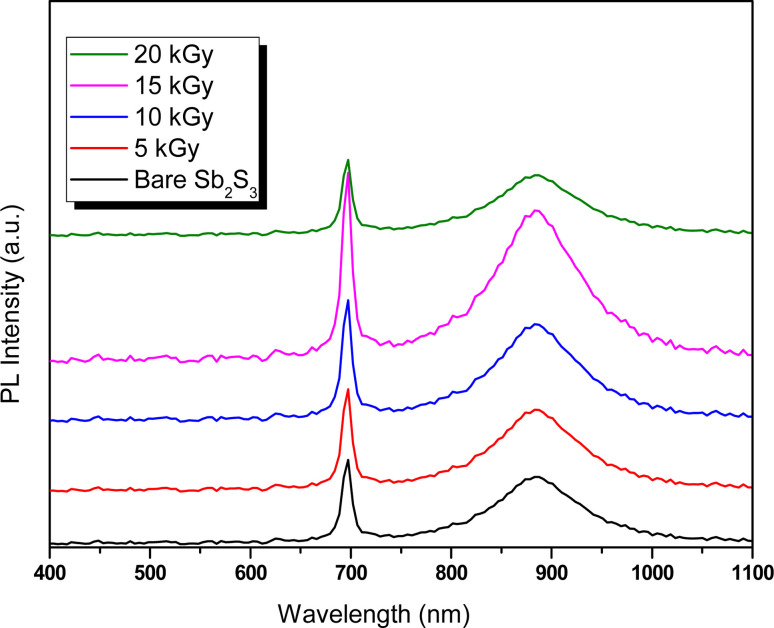
Photoluminescence (PL) spectra of Sb_2_S_3_ thin films before and after γ-irradiation with different doses.

Photoluminescence (PL) is a non-destructive technique for probing defects and electronic properties in semiconductor materials. Fig. 13 shows the dose-dependent PL emission spectra for bare and γ-irradiated Sb_2_S_3_ films scrutinized with an excited laser wavelength of 266 nm. The PL emission spectra of bare film showed a narrow band with a peak around 696 nm (corresponding to 1.78 eV) and a wide emission peak at 885 nm wavelength (corresponding to 1.40 eV). This first band is attributed to the band-to-band emission of Sb_2_S_3_, roughly in line with the conclusions drawn from UV-VIS spectroscopic measurements, while the second band could be ascribed to the state of vacancies or defects in Sb_2_S_3_ films.^67^ Recently, Krautmann *et al.* have reported a similar variation of room temperature PL analysis of the synthesized Sb_2_S_3_ films by close-spaced sublimation, where they found two PL peaks located at 1.72 eV and 1.40 eV.^68^ Meanwhile, Aslan *et al*. performed PL analysis for dip-coating Sb_2_S_3_ films photodetector. Therein the authors have found three PL emissions peaks located at 400, 521, and 652.5 nm.^69^ The slim contrast in the values of the PL emission peaks would be attributed to lattice distortion caused by γ rays. On the other hand, we note the increase in PL intensity with increasing irradiation doses. Especially, the film exposed to a dose of 15 kGy exhibits the greatest PL intensity, which is 2.2 orders of magnitude greater than that of the unexposed film. The increase in PL intensity upon irradiation could be due to the fusion and agglomeration of particles, resulting in the formation of larger grains, which can effectively annihilate the surface defects and consequently reduce non-radiative recombination losses and raise radiative emission. Furthermore, it should be noted that a broadening of γ-irradiated thin films compared to bare Sb_2_S_3_ sample is observed due to inhomogeneous particle size and to self-trapped exciton states induced by the lattice distortions and surface defects. At 20 kGy, a reduced PL intensity is observed compared to the other samples, suggesting increased non-radiative recombination, although this sample has the lowest FWHM.

### 3.7 Wettability analysis

**Fig. 14:**
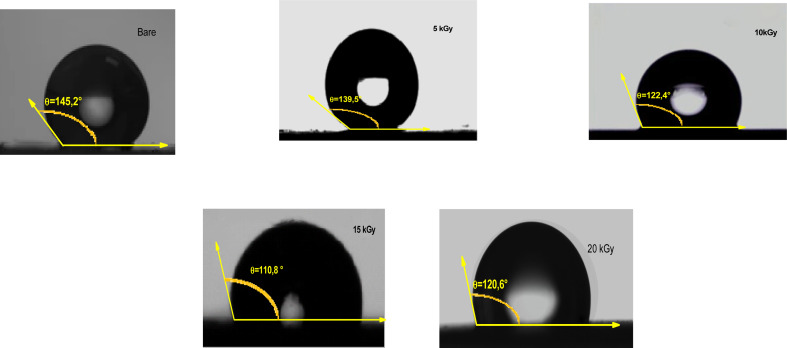
Water contact angle of as-deposited and γ-irradiated Sb_2_S_3_ thin films with different doses.

Wettability tests were used to measure the ability of a water droplet to spread and adhere to a solid surface. The CA formed by a drop on a solid surface is a crucial parameter for evaluating wettability and can be influenced by various factors, including surface tension, surface roughness, and chemical composition.^70^ A low CA (close to 0°) indicates high wettability or hydrophilicity, while a high CA (close to 180°) indicates low wettability or hydrophobicity. As a matter of fact, γ radiations can alter the CA and surface wetting behavior, which is a crucial aspect for efficient H_2_ production in aqueous solution. In this context, the surface wettability of the Sb_2_S_3_ thin film was investigated at various γ-irradiation doses. As illustrated in Fig. 14, all the samples exhibit a CA greater than 90°, suggesting low wettability and consequently resulting in a low Gibbs surface free energy.^71^ The CA decreases from 145.2° to 110.8° with increasing gamma irradiation, which may affect the electrolyte absorption and the interaction between Sb_2_S_3_ photoelectrodes and electrolytes. The optimal CA was noted for the sample irradiated with a dose of 15 kGy, signifying a higher surface wettability. The observations also agree with the results of the AFM measurements. In fact, lower CA corresponds to higher surface roughness. It is known that chalcogenide materials typically consist of elements with similar electronegativity that form a nonpolar covalent bond, resulting in a hydrophobic surface.^72^ These hydrophobic properties with rough surfaces generated by γ irradiations tend to trap air pockets, impeding complete wetting by the water drop. This obtained low CA has a positive effect on improving the adsorption surface between the Sb_2_S_3_ thin film and the water drop. An enhancement in PEC performance is expected for the specific film treated with a dose of 15 kGy, as this condition allowed further improvement in surface hydrophilicity.

### 3.8 Photoelectrochemical measurements

**Fig. 15:**
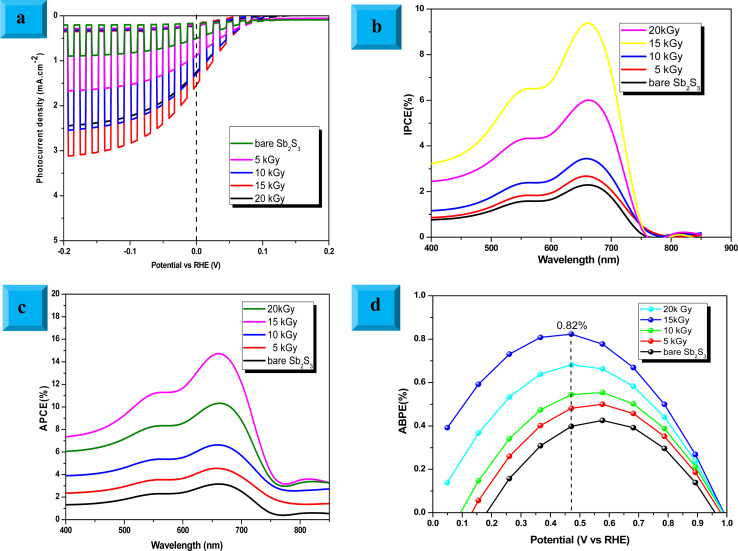
(a) Linear sweep voltammetry (LSV) plots of the bare and irradiated Sb_2_S_3_-based photocathodes. (b) The incident photocurrent conversion efficiencies (IPCEs) spectra were recorded for Sb_2_S_3_-based photocathodes with an applied bias voltage of 0 V *vs.* RHE under front illumination. (c) Absorbed photon-to-current efficiency (APCE) spectra were recorded for all Sb_2_S_3_-based photocathodes. (d) Calculated applied bias photon-to-current efficiency (ABPE) for all Sb_2_S_3_-based photocathodes. All measurements were carried out in 0.5 M Na_2_SO_4_ buffer (pH ≅ 7) electrolyte.

**Fig. 16:**
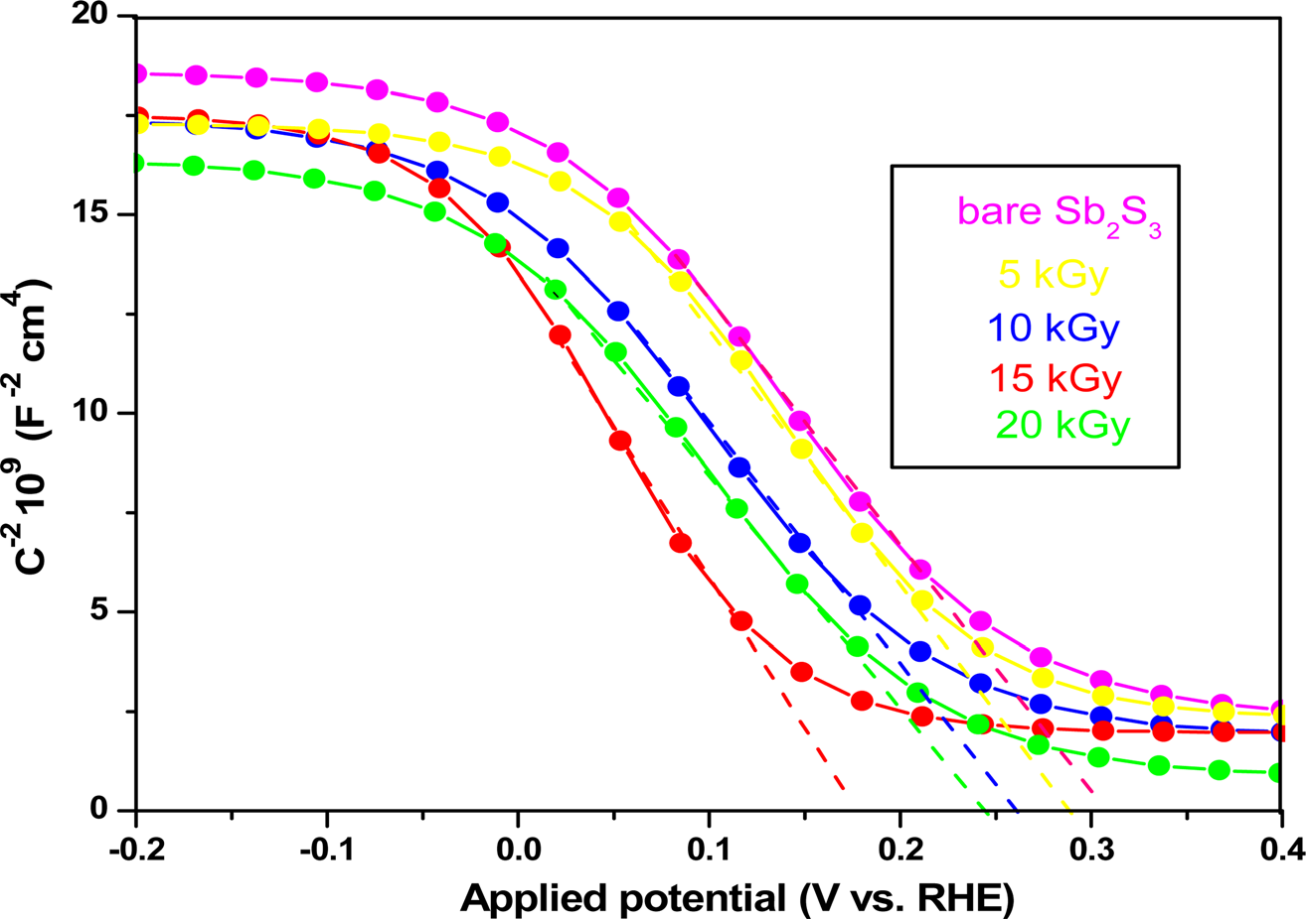
Mott–Schottky plots of bare and irradiated Sb_2_S_3_-based photocathodes.

**Fig. 17:**
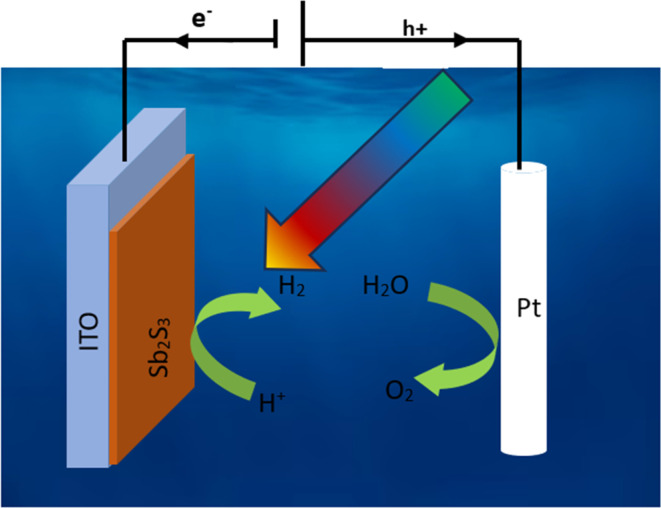
Schematic mechanism of photoelectrochemical water splitting of Sb_2_S_3_-based photocathode.

**Fig. 18:**
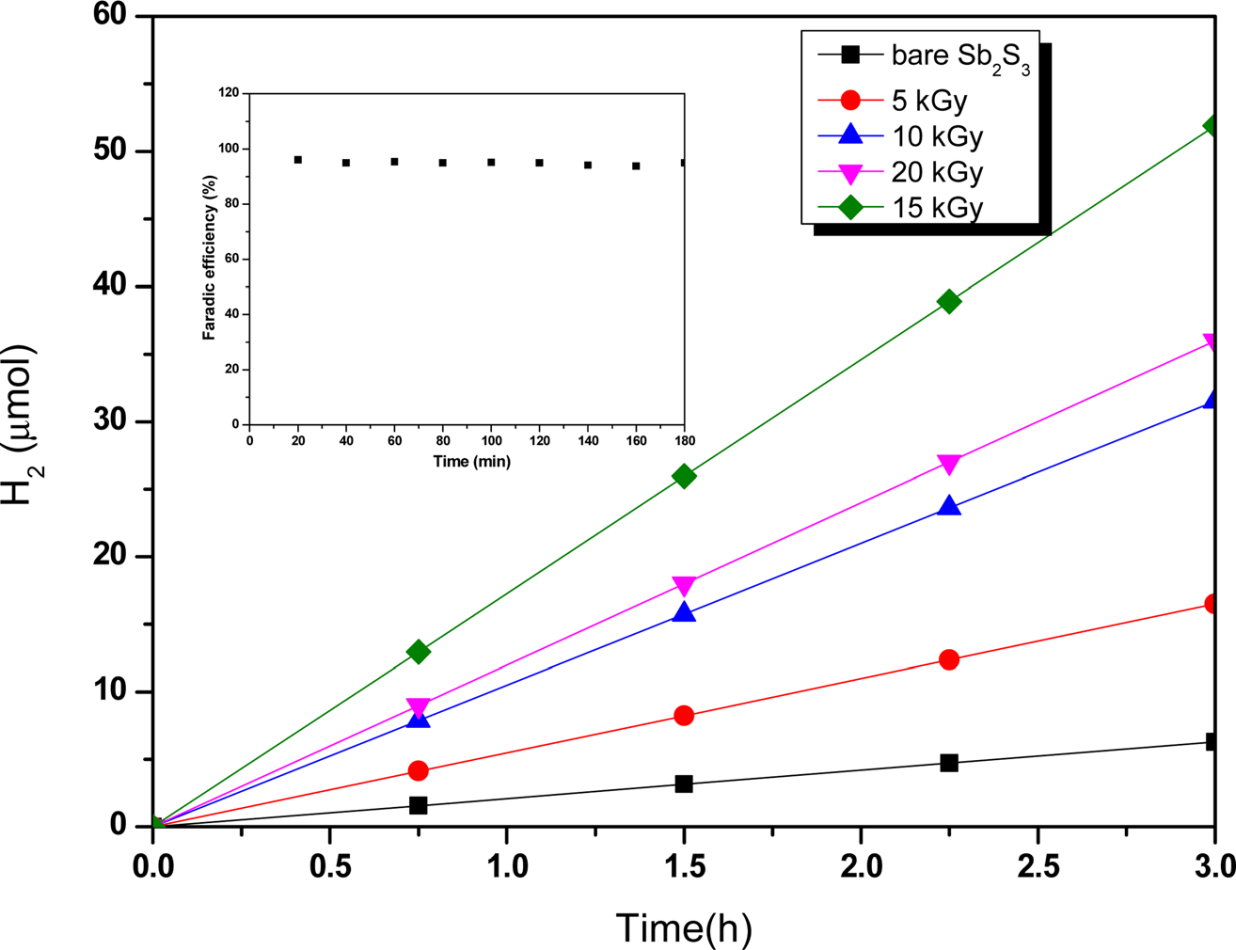
Hydrogen evolution as a function of time for different Sb_2_S_3_-based photocathodes under standard one sun (100 mW cm^−2^) illumination at 0 V *vs.* RHE. The inset plot of Fig. 15 stands for the faradaic efficiency (FE) measurement of the optimized Sb_2_S_3_-based photocathode (15 kGy).

To elucidate the effect of γ radiations on the PEC properties of Sb_2_S_3_ photoelectrodes, photocurrent density–voltage (*J*–*V*) tests were carried out under chopped white LED illumination using a standard three-electrode cell in an aqueous sodium sulfate solution (Na_2_SO_4_) with a concentration of 0.5 mol L^−1^ and a pH ≅ 7. The active surface area of the working electrode was set at *ca.* 0.16 cm^2^, and the counter electrode used was Pt. Fig. 15a exhibits representative chopped (dark/light) linear sweep voltammetry (LSV) scans of bare and irradiated Sb_2_S_3_ samples from −0.2 to 0.2 V *vs.* RHE with a periodic time of 1 s light on/off. Since there is a negligible current density on all photoelectrodes in dark lighting conditions, this is not displayed. By the way, no anodic transient current was detected during the entire measurement period that would be responsible for back electron transfer when the light was turned off. In striking contrast, all Sb_2_S_3_ photoelectrodes produced cathodic photocurrents upon illumination, corroborating the p-type conductivity of Sb_2_S_3_ films determined by Hall effect measurement. The onset potentials of all photocathodes were approximately 0.15 V *vs.* RHE. For comparison, other encouraging photocathodes like Cu_2_ZnSnS_4,_ CuO, and Si typically exhibit a photocurrent onset at around 0.7 V *vs.* RHE for water reduction.^73^ However, similarly high photocurrent onset potentials have recently been stated for CuBi_2_O_4_ and Cu_2_O.^74^ During the intermittent illumination periods, there is very little leakage current, as demonstrated by the noticeable increase and decrease in photocurrent density upon exposure to light.^22^ The obtained results of the transient photocurrent response certainly corroborated that the photocurrent density generated from the irradiated Sb_2_S_3_ photocathodes was meaningfully higher than that of the bare photocathode owing to the existence of a charge transport mechanism in the irradiated Sb_2_S_3_ samples. The bare sample shows the lowest transient photocurrent responses of *ca.* 0.60 mA cm^−2^ at 0 V *vs.* RHE, indicating the highest recombination rate of photogenerated excitonic pairs, as well as the significantly inadequate electron mobility in the Sb_2_S_3_ film. The obtained photocurrent density of the bare photocathode aligns with findings from other reports.^75^ Comparatively, Sb_2_S_3_ subjected to a dose of 15 kGy shows the strongest photo-current density, reaching a value of 1.62 mA cm^−2^ at 0 V *vs.* RHE. This magnitude is almost 2.7 times higher than that of the bare sample, indicating a notable enhancement in the efficiency of photogenerated charge separation. The rise in photocurrent density is attributed to the increased number of sulfur vacancies generated by γ radiations, which facilitates the capture of electrons from the conduction band of Sb_2_S_3_ and thus improves the charge separation ability. The introduction of vacancies in semiconductors could have multiple effects on their properties. Indeed, vacancies can improve photo absorption capabilities, acting as traps for photogenerated electrons, which helps limit the recombination of excitonic pairs.^76^ This phenomenon contributes to enhanced photo reactivity in semiconductor materials. However, a higher irradiation dose (>15 kGy) may create more bulk vacancies, resulting in a lower photocurrent density. These defects and traps within the photocathode acted as recombination centers and led to a reduction in photocurrent density. To assess the charge separation efficiency of the irradiated Sb_2_S_3_-based photocathodes in comparison to the bare one, the IPCE and APCE of all photocathodes were then measured in the wavelength spectrum ranging from 400 nm to 900 nm under front illumination with an applied bias voltage of 0 V *vs.* RHE. The wavelength dependence of the IPCE and APCE of Sb_2_S_3_-based photocathodes with different γ radiation doses is shown in Fig. 15b and c. The IPCE and APCE values of the photocathode irradiated with 15 kGy are significantly higher than the other photocathodes. They exhibit an IPCE peak and an APCE peak at 648 nm, with values of 9.35% and 14.47%, respectively. This means that the optimized sample has a higher quantum efficiency than the other ones. The dependence of the IPCE and APCE performance on the irradiation dose is attributed to the band gap of the irradiated films since significant variations were also found in the absorption spectra. This correlation can be attributed to the ease of transporting photocarriers through the multilayers in this Sb_2_S_3_ structure, as the enhancement in conversion efficiency depends on the absorption of the incident photons and the transmission of the photogenerated carriers to reach the surface for the chemical reaction. A possible reason for the loss of efficiency above a dose of 15 kGy is ascribed to the low crystallinity of Sb_2_S_3_ films and imperfect interfaces, which lead to high recombination rates. The limited photoconversion performance in terms of IPCE and APCE at *λ* over 756 nm is consistent with the optical band gap of irradiated Sb_2_S_3_ semiconductors, which primarily absorb light to generate electrons and holes for the PEC charge transfer reaction across their surface. What is more, to fully evaluate the PEC water-splitting performance of all photocathodes, we measured the ABPE, which is related to the amount of energy used during the process.^77^Fig. 15d displays the APBE of the synthesized photocathodes. The maximum efficiency of the 15 kGy-irradiated sample was 0.82% at 0.47 V *vs.* RHE, which was significantly lower than other irradiated samples. 15 kGy-irradiated sample exhibited the lowest potential and higher efficiency, suggesting superior PEC water splitting performance compared to other samples. The enhanced ABPE of the optimized sample is due to the boosted visible light resulting in improved exciton dissociation and transfer. It is worth noting that the optimized photocathode increases the conversion efficiency while decreasing the required potential to achieve the maximum photoconversion efficiency. Meanwhile, to acquire a deeper understanding of the charge transfer and separation processes in all Sb_2_S_3_ samples, M–S analysis was conducted. The carrier concentration and flat band potential (*V*_fb_) were estimated from the slope of a plot of 1/*C*^2^*vs.* V, and the *x*-intercept of the extrapolated linear segment of the M–S plot, respectively. The calculation was based on the following relationship:12
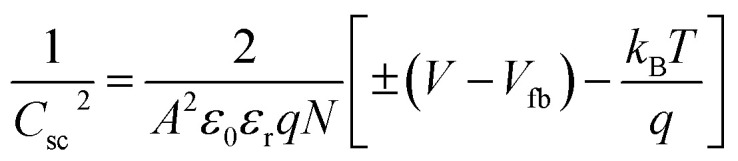
where *C*_sc_ is space charge capacitance (F), *q* is an elementary charge (1.6 × 10^−19^ C), *A* is electrode surface area (cm^2^), *ε*_r_ = 6.67 is the relative permittivity of Sb_2_S_3_,^78^*ε*_0_ is vacuum permittivity (8.854 × 10^−12^ F m^−1^), *N* is carrier concentration in the semiconductor (cm^−3^), *V* is applied potential (V), *V*_fb_ is flat band potential (V), and *k*_B_ is Boltzmann constant (1.38 × 10^−23^ J K^−1^), and *T* is the absolute temperature (K). 
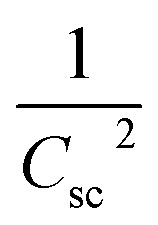
 decreases with the applied bias voltage in the presence of a space charge region, confirming the p-type semiconductor behavior for all samples, as shown in Fig. 16. In addition, γ irradiation does not affect the p-type character of the Sb_2_S_3_ semiconductor, echoing the aforementioned (*J*–*V*) measurements. The result obtained is in good agreement with the findings from previous research studies.^79^ In addition, the acceptor density (*N*_A_) estimated from the slopes of M–S plots increases from 2.8 × 10^15^ cm^3^ (bare film) to 4.6 × 10^15^ cm^3^ (15 kGy irradiated film) and then decreases to 3.1 × 10^15^ cm^3^ when the irradiation doses reach 20 kGy. The changes in acceptor density can be attributed to the sulfur vacancies caused by γ-irradiation. The 15 kGy films exhibited a significantly higher acceptor density compared to the bare sample. This increase occurred despite no major changes in the phase structure of the Sb_2_S_3_ material. However, there is a slight discrepancy between the acceptor density determined by the Hall effect and Mott–Schottky measurements that could be attributed to sensitivity to surface effects, sample homogeneity, and band bending. The derived *V*_fb_ of the bare and irradiated Sb_2_S_3_ films at 5, 10, 15, and 20 kGy were 0.31, 0.29, 0.27, 0.17, and 0.24 V *vs.* RHE, respectively. It was gradually decreased along with the increased irradiation doses up to 15 kGy, while it was increased around the value of 0.24 V *vs.* RHE for the irradiation dose of 20 kGy. In other words, the band bending at the photocathode/electrolyte interface is strengthened by increasing the irradiation doses up to 15 kGy, while it decreases when the irradiation doses reach 20 kGy. By the way, the Mott–Schottky curve exhibits nonlinearity in the potential range from −0.2 to 0.4 V *vs.* RHE. This behavior could be attributed to various factors related to the Sb_2_S_3_/Na_2_SO_4_ interface, such as interface roughness and/or surface states at the Sb_2_S_3_ samples.^80^ The downward shift of the *V*_fb_ value toward the valence band indicates a decrease in the Fermi level, leading to an increase in the hole concentration of the Sb_2_S_3_ photocathode. Based on these results, the optimized photocathode (15 kGy irradiated film) enables more efficient electron transfer between the Sb_2_S_3_/Na_2_SO_4_ interface. Thus, the higher photocurrent revealed by the optimized photocathode could be explained by the high concentration and separation efficiency of the photogenerated species, resulting in improved PEC performance. Fig. 17 illustrates the mechanism of the PEC process, wherein photogenerated species encounter water molecules in the electrolyte after absorbing a photon. The electrons traverse through the conduction band of the working electrode and directly react with two protons in the Na_2_SO_4_ buffer electrolyte to produce H_2_, while the holes traverse through the external wire circuit until they reach the Pt electrode. There, they react with two H_2_O molecules to generate O_2_. The primary event that induces the observed photocurrents is likely water reduction to H_2_ gas. To quantify the amount of H_2_ in the gas phase, H_2_ evolution across all Sb_2_S_3_ photocathodes was measured *via* micro gas chromatography (GC) using a gas-tight syringe as the delivery tool. Fig. 18 exhibits the amount of H_2_ evolved from a unit area of all Sb_2_S_3_ photocathodes *versus* time under standard one sun (100 mW cm^−2^) illumination at 0 V *vs.* RHE. We have not included the error bar here since each calculated gas evolution rate is the measurement of 2–3 GC tests. We have used the average PPM (parts per million) to estimate the rate of hydrogen evolution. It is worth noting that the linear relationship shows the excellent stability of the synthesized samples. On the other hand, the bare sample displays poor hydrogen evolution activity of around 2.07 μmol h^−1^ owing to the limited light absorption, which was in good agreement with the previous literature reports.^81^ According to Xiao *et al.*^82^ this phenomenon could be explained by the effect of overvoltage for the hydrogen evolution activity, which requests the bandgap value of the semiconductor material to be over 1.8 eV, while that of bare Sb_2_S_3_ was only 1.78 eV. However, the hydrogen evolution activity of irradiated samples increased, demonstrating that the irradiated Sb_2_S_3_ samples perform efficient photocatalytic reactions under visible light. The evolution rate of H_2_ increases with increasing γ radiation doses and attains a maximum of 17.20 μmol h^−1^ at 15 kGy, which is about 8.3 times higher than that of the bare sample. Thereafter, further increasing the γ radiation doses led to a significant reduction in photoactivity, which is evident from the lower H_2_ evolution rate of the 20 kGy sample (9.7 μmol h^−1^). To establish the total volume of hydrogen gas produced during 180 minutes of the best optimized films, the FE was determined through a comparative analysis of the detected volume of H_2_ gas and the calculated volumes of H_2_ gas with a theoretical 100% FE, as shown in the inset of Fig. 18. During about 180 minutes, 9.0 mL of H_2_ with an FE of nearly 95.36% was achieved. The remaining 4.64% could be due to ineffective gas collection and/or some parasitic electrochemical processes. Such a phenomenon has also been demonstrated in another PEC water-splitting system.^83^ We have conclusively demonstrated that the 15 kGy photocathode is highly effective for PEC water splitting to produce H_2_ gas. However, ongoing efforts are focused on further improving the quality of the Sb_2_S_3_ semiconductor and optimizing processes for surface adjustments.

## 4. Conclusions

In summary, the introduction of sulfur vacancies into Sb_2_S_3_ thin films through γ-irradiation was carried out to enhance its PEC performance for hydrogen production. γ-irradiation led to an improved crystal structure, reduction in the band gap energy, and altered morphology of Sb_2_S_3_ film, resulting in suppressed recombination of photogenerated carriers and enhanced charge transportation. Moreover, the irradiation process produced an increase in Sb_2_S_3_ film conductivity. The PEC water-splitting analysis discloses that the film treated under an irradiated dose of 15 KGy photocathode boosted the photocurrent density from 0.60 mA cm^−2^ to 1.62 mA cm^−2^ at 0 V *vs.* RHE (2.7-fold enhancement) compared with bare Sb_2_S_3_. The significant enhancement in PEC performance is attributed to various factors, including crystallite size, surface roughness, low wettability, and band gap. Hence, γ-irradiation offers a novel strategy for controlling the surface vacancies of Sb_2_S_3_ films, leading to high PEC performance. The treatment with γ-irradiation showed outstanding performance in photo-generated charge transfer and separation. Our outstanding results open a new avenue that highlights the energetic influence of γ-irradiation on Sb_2_S_3_ thin films and enables the construction of highly stable and efficient photocathodes for solar-driven PEC water-splitting applications.

## Conflicts of interest

The author confirms that there are no known competing financial interests or personal relationships associated with this publication for this work that could have influenced its outcome.

## Supplementary Material
